# Brivaracetam, but not ethosuximide, reverses memory impairments in an Alzheimer’s disease mouse model

**DOI:** 10.1186/s13195-015-0110-9

**Published:** 2015-05-05

**Authors:** Haakon B Nygaard, Adam C Kaufman, Tomoko Sekine-Konno, Linda L Huh, Hilary Going, Samantha J Feldman, Mikhail A Kostylev, Stephen M Strittmatter

**Affiliations:** Department of Neurology, Yale University School of Medicine, 800 Howard Avenue, New Haven, CT 06510 USA; Cellular Neuroscience, Neurodegeneration, and Repair Program (CNNR), Yale University School of Medicine, 295 Congress Avenue, New Haven, CT 06536 USA; Division of Neurology, The University of British Columbia, Djavad Mowafaghian Centre for Brain Health, 2215 Wesbrook Mall, Vancouver, BC V6T 1Z3 Canada; Division of Pediatric Neurology, The University of British Columbia, British Columbia Children’s Hospital, 4480 Oak Street, Vancouver, BC V6H 3V4 Canada

## Abstract

**Introduction:**

Recent studies have shown that several strains of transgenic Alzheimer’s disease (AD) mice overexpressing the amyloid precursor protein (APP) have cortical hyperexcitability, and their results have suggested that this aberrant network activity may be a mechanism by which amyloid-β (Aβ) causes more widespread neuronal dysfunction. Specific anticonvulsant therapy reverses memory impairments in various transgenic mouse strains, but it is not known whether reduction of epileptiform activity might serve as a surrogate marker of drug efficacy for memory improvement in AD mouse models.

**Methods:**

Transgenic AD mice (APP/PS1 and 3xTg-AD) were chronically implanted with dural electroencephalography electrodes, and epileptiform activity was correlated with spatial memory function and transgene-specific pathology. The antiepileptic drugs ethosuximide and brivaracetam were tested for their ability to suppress epileptiform activity and to reverse memory impairments and synapse loss in APP/PS1 mice.

**Results:**

We report that in two transgenic mouse models of AD (APP/PS1 and 3xTg-AD), the presence of spike-wave discharges (SWDs) correlated with impairments in spatial memory. Both ethosuximide and brivaracetam reduce mouse SWDs, but only brivaracetam reverses memory impairments in APP/PS1 mice.

**Conclusions:**

Our data confirm an intriguing therapeutic role of anticonvulsant drugs targeting synaptic vesicle protein 2A across AD mouse models. Chronic ethosuximide dosing did not reverse spatial memory impairments in APP/PS1 mice, despite reduction of SWDs. Our data indicate that SWDs are not a reliable surrogate marker of appropriate target engagement for reversal of memory dysfunction in APP/PS1 mice.

**Electronic supplementary material:**

The online version of this article (doi:10.1186/s13195-015-0110-9) contains supplementary material, which is available to authorized users.

## Introduction

Despite significant advances in the understanding of Alzheimer’s disease (AD), an effective disease-modifying intervention has not yet been identified. It is now well established that patients with AD have an increased risk of seizures [1]. In sporadic AD, the frequency of seizures vary considerably between studies, with more recent reports estimating an incidence of approximately 4 to 5 per 1,000 persons per year [2,3]. Epilepsy is common in familial AD, with an incidence as high as 83% in these patients [1]. Several groups, including ours, have shown that mice overexpressing the amyloid precursor protein (APP) also have seizures [4-6]. These findings have led to the hypothesis that amyloid-β (Aβ), the peptide derived from APP and widely believed to play a critical role in AD pathogenesis, may trigger neuronal hyperexcitability, seizures, and ultimately worsen neuronal dysfunction in AD. This hypothesis was partly tested in two recent studies where transgenic AD mice underwent chronic treatment with the antiepileptic drug (AED) levetiracetam [7,8]. In the initial report, treatment with levetiracetam was described as strongly reducing epileptiform discharges (single spikes), ameliorating memory impairments and reversing markers of hyperexcitability, including calbindin D28 and neuropeptide Y. The same drug was recently shown to improve select hippocampal function in human subjects diagnosed with amnestic mild cognitive impairment (aMCI) [9], suggesting a potential therapeutic benefit of levetiracetam in aMCI and possibly AD.

The mechanisms underlying the improvements seen in AD mice treated with levetiracetam are presumed to involve a reduction in neuronal excitability, and, although this hypothesis has not been directly tested, targeting epileptiform discharges has emerged as a potential therapeutic approach in AD [7,10]. This is supported by recent work showing that a genetic reduction in either endogenous tau protein or cellular prion protein (PrP^C^), both of which reverse impairment in spatial memory in AD mice, is associated with a reduction in aberrant neuronal activity in rodent models of AD [6,11,12]. These findings would suggest that a reduction in epileptiform discharges can predict a therapeutic reversal in spatial memory impairments, with reduced neuropathology, in transgenic AD mice. This would be important because behavioral testing in mice, still considered an important step in preclinical drug development, requires significant time and resources that could be optimized by availability of a reliable surrogate marker of drug efficacy.

Using continuous *in vivo* electroencephalography (EEG) recording, coupled with spatial memory testing, we studied whether epileptiform discharges in transgenic AD mice could be used as a marker of drug efficacy for memory improvement. We report that in two transgenic AD models, APP/PS1 [13] and 3xTg-AD [14], the presence of spike-wave discharges (SWDs) correlate with impairments in spatial memory, although a weaker correlation was seen in 3xTg-AD mice. Biochemical and immunohistochemical analyses indicated that these epileptiform discharges were not associated with changes in Aβ metabolism or deposition. We further demonstrate that SWDs can be suppressed by the AEDs ethosuximide and brivaracetam, with no effect seen when phenytoin was used. Interestingly, brivaracetam, but not ethosuximide, reversed memory deficits in APP/PS1 mice, despite both drugs causing a strong reduction in epileptiform discharges. Our data indicate that SWDs are associated with poor cognitive performance in APP/PS1 mice, but that the reduction of this abnormal network activity does not reliably predict therapeutic reversal of age-associated impairments in spatial memory in this mouse model. We confirm that targeting synaptic vesicle protein 2A (SV2A), which results in broad-spectrum anticonvulsant action, reverses memory impairments in the APP/PS1 model of AD.

## Methods

### Mice

The use of mice in this study was approved by the Yale Animal Resources Center according to internationally recognized guidelines. All mice were housed with a 12-hour light/12-hour dark cycle and fed *ad libitum*. Coinjected congenic APPswe/PSEN1dE9 transgenic mice [13] on a pure C57BL/6J background were obtained from The Jackson Laboratory (Bar Harbor, ME, USA). 3xTg-AD mice were a gift from Dr Frank LaFerla (UC Irvine, CA, USA) and were obtained via Dr Paul Lombroso (Yale University). They express the mutated knockin gene PS1M146V, as well as APPswe and tauP301L, at the same locus, both under control of the mouse Thy1.2 regulatory element [14]. 3xTg-AD mice were on a mixed C57BL/6J × 129/Sv background as described elsewhere [14]. For chronic drug experiments, mouse groups were sex-matched, with 40% to 60% of each sex in different cohorts.

### Behavioral studies

For experiments correlating epileptiform activity, animals underwent EEG monitoring prior to behavioral testing. Animals tested for behavior during treatment with AEDs did not have EEG electrodes implanted. Animals were randomized, and the experimenter was blinded to genotype for the duration of behavioral testing. Morris water maze testing [15] based on previously described methods [12] was performed over the course of 3 days. Each swim was performed at room temperature in an open-water pool approximately 1.3 m in diameter, utilizing a submerged, nonvisible escape platform located in the center of one of the pool’s four quadrants. This location remained constant for the 3 days of testing. Over the course of each testing day, an animal swam a total of eight times—four times in the morning, constituting one “block” of swims, and four times in an afternoon block. The interval between blocks was approximately 2 hours. For each block, the mice would begin their swim in one of four distinct locations around the wall of the pool and were timed for its latency and path length to reach the escape platform for a maximum time of 1 minute. If the mouse did not find the submerged platform by 1 minute, it was placed on the platform for approximately 10 seconds before being removed from the pool. The water maze probe trial was performed 48 hours following the third and last day of the memory acquisition phase and in the same 1.3-m pool described above. For the purposes of the probe trial, the platform was removed from the pool. All mice were started from a location opposite to the platform location and allowed to swim for 1 minute. To ensure that all mice were equal in terms of swim speed, motivation and visual acuity, a block of five swims to a visible platform was conducted after the probe trial. Mice were excluded from the study if the latency to the visible platform exceeded 3 standard deviations above the average latency for control mice, as previously described [16]. By this criterion, one APP/PS1 mouse with SWDs was excluded. Latency to the platform, swim speed, path length and resting time were automatically recorded using Panlab SMART video tracking and analysis program, v2.5 (Panlab, Cornellà de Llobregat, Spain).

### Brain tissue collection

Mice were deeply anesthetized with isoflurane and immediately perfused with ice-cold 0.9% NaCl for 2 minutes. Their brains were then dissected out and placed in ice-cold 0.9% NaCl. For biochemical analysis, the right hemibrain was weighed and immediately frozen in liquid nitrogen, followed by storage at −80°C. To extract the soluble cytosolic fraction, the brains were homogenized in 3 volumes (w/v) of 50 mM Tris-HCl, 150 mM NaCl, pH 7.6 (TBS), containing a protease inhibitor cocktail (cOmplete Protease Inhibitor Cocktail, catalog number 10745000; Roche Diagnostics, Mannheim, Germany), 1 mM sodium orthovanadate and 50 mM sodium fluoride. Tissue was homogenized using an ultrasonic cell disruptor (Branson Ultrasonics Corporation, Danbury, CT, USA) and ultracentrifuged at 100,000 × *g* for 20 minutes at 4°C. The pellet was then resuspended to the same volume as the original homogenate in TBS with 2% Triton X-100 (AmericanBio, Natick, MA, USA), 0.1% SDS (AmericanBio), a protease inhibitor cocktail (cOmplete Protease Inhibitor Cocktail), 1 mM sodium orthovanadate and 50 mM sodium fluoride. Tissue was homogenized and ultracentrifuged at 100,000 × *g* for 20 minutes. The supernatant was mixed with 4× SDS-PAGE loading buffer, boiled for 5 minutes and stored for subsequent analysis.

### Immunohistochemistry

One hemibrain was immersed in fresh 4% paraformaldehyde (PFA) overnight. After the brains were fixed, they were embedded in 10% gelatin and placed in 4% PFA for 20 hours at 4°C. Parasagittal sections (30 μm) were then cut using a Leica VT1000 S vibratome (Leica Biosystems, Buffalo Grove, IL, USA). For immunohistochemistry, sections were blocked in 10% donkey serum for 1 hour, followed by incubation with primary antibody overnight at room temperature. Primary antibodies were diluted in phosphate-buffered saline (PBS) with 0.2% Triton X-100 (AmericanBio). The following antibodies were used: Aβ antibody (catalog number 2454, Cell Signaling Technology, Danvers, MA, USA; and clone 6E10, monoclonal antibody 1560, EMD Millipore, Billerica, MA, USA: both diluted 1:250), rabbit anti-PSD-95 polyclonal antibody (1:250 dilution, catalog number 51-6900; Invitrogen, Camarillo, CA, USA) and anti-calbindin D28 antibody (1:1,000 dilution; Swant, Marly, Switzerland). Following incubation, the sections were washed three times with PBS and incubated in Alexa Fluor fluorescent secondary antibody (donkey anti-rabbit or anti-mouse, all at 1:500 dilution; Invitrogen) for 2 hours at room temperature. The slices were then washed three times and transferred to PBS. Sections were also stained with secondary antibody alone to rule out nonspecific staining. Each free-floating section was mounted on a microscope slide (Fisherbrand Superfrost Plus; Fisher Scientific, Pittsburgh, PA, USA) and coverslipped using VECTASHIELD mounting medium (H-1000; Vector Laboratories, Burlingame, CA, USA).

### Imaging and analysis

All images and analyses were generated by personnel who had no knowledge of the mouse genotype. Aβ images were obtained using a Zeiss Axio Imager Z1 fluorescence microscope (Carl Zeiss Microscopy, Jena, Germany) with a 10× lens objective. Mosaic images of the entire cortex and hippocampus of each animal were obtained and analyzed, and plaque burden was calculated using ImageJ software (National Institutes of Health, Bethesda, MD, USA). This was done by isolating the cortex or hippocampus, thresholding to a standard value and calculating the area occupied. Using an Ultra*VIEW* VoX spinning disc confocal microscope (PerkinElmer, Waltham, MA, USA), hippocampal PSD-95 immunoreactive puncta were imaged with a 60× lens objective and digitally magnified to × 100. Two images were obtained in the molecular layer of the dentate gyrus with two slices from each mouse analyzed. Puncta from the dentate gyrus were analyzed and counted using ImageJ, excluding cell somata. Hippocampal calbindin D28 images were obtained using a Zeiss Axio Imager Z1 fluorescence microscope with a 20× lens objective. Mosaic images of the entire hippocampus of each animal were obtained and analyzed. All histologic analyses were done using ImageJ and analyzed statistically by Student’s *t*-test, or by analysis of variance (ANOVA) with *post hoc* comparisons as indicated, using SPSS software (IBM, Armonk, NY, USA).

### Immunoblotting and enzyme-linked immunoassay experiments

Precast 10% Tris-glycine or 10–20% Tris-tricine gels were used (Bio-Rad Laboratories, Hercules, CA, USA). After transfer, the polyvinylidene fluoride membranes (catalog number 162-0174; Bio-Rad Laboratories) were incubated in blocking buffer for 1 hour at room temperature (catalog number 927-4000, Odyssey blocking buffer; LI-COR Biosciences, Lincoln, NE, USA). Membranes were then washed five times in a mixture of Tris-buffered saline and Tween 20 (TBST) and incubated overnight in primary antibodies. The following antibodies were used: clone 6E10 (MAB1560, 1:1,000 dilution; EMD Millipore), clone 22C11 (MAB348, 1:100 dilution; EMD Millipore) and actin (catalog number sc-1616, 1:200 dilution; Santa Cruz Biotechnology, Santa Cruz, CA, USA). All antibodies were diluted in Odyssey blocking buffer, and membranes were incubated overnight at 4°C. Following primary antibody incubation, the membranes were washed five times with TBST, and secondary antibodies were applied for 1 hour at room temperature (Odyssey donkey anti-mouse or anti-goat IRDye (LI-COR Biosciences) at 680 or 800 nm). Membranes were then washed, and proteins were visualized using a LI-COR Odyssey Infrared imaging system. Blots were analyzed using ImageJ and normalized to actin optical density. Total Aβ enzyme-linked immunosorbent assay (ELISA) experiments on TBS-soluble mouse brain lysates were performed according to the manufacturer’s instructions (Invitrogen).

### Continuous electroencephalography video monitoring

For dural electrode implantation, the mice anesthetized and maintained with inhaled isoflurane and mounted in a stereotaxic frame (David Kopf Instruments, Tujunga, CA, USA). A midline incision was made, and two bilateral burr holes were manually drilled anterolateral and posterolateral to the bregma. Four presoldered intracranial screw electrodes (catalog number 8403; Pinnacle Technology, Lawrence, KS, USA) or a prefabricated headmount (catalog number 8201; Pinnacle Technology) was inserted and secured with a layer of dental cement (catalog number 526000; A-M Systems, Sequim, WA, USA). In the case of presoldered screw electrodes, the electrode wires were soldered onto a six-pin surface mount connector (catalog number 8235-SM; Pinnacle Technology) and secured by a final layer of dental cement. All mice were allowed to recover for 7 days prior to chronic EEG recordings.

Mice were video-recorded using an *in vivo* EEG video monitoring system (8200-K1-SE3, 8236; Pinnacle Systems). EEGs were sampled at 400 Hz with 100× preamplifier gain and filtered at 30 Hz. Each mouse underwent 72 hours of continuous EEG video recording and was maintained on a regular 12-hour light/12-hour dark cycle with full access to food and water. EEG traces were scored manually by an investigator, blinded to genotype, using Pinnacle Technology software. A convulsive seizure was defined as an abrupt onset of evolving SWDs lasting >30 seconds, associated with tonic-clonic activity by synchronized video analysis, and followed by postictal attenuation of cerebral EEG rhythms. A SWD was defined as a burst of sharp-wave discharges, with an amplitude of at least twice the background amplitude and 1 to 2 seconds in duration. A single spike was defined as a sharp discharge at least twice the background amplitude and <100 milliseconds in duration. SWDs were manually correlated with synchronized video analysis and scored as with or without behavioral arrest. Twenty-four-hour EEGs were manually scored for single spikes. A full 72 hours of EEG were manually screened for SWDs for each mouse, comprising 24 hours before drug delivery and 48 hours afterward. Epileptiform discharges were analyzed using Student’s *t*-test.

### Drug administration

Each mouse received a single intraperitoneal (IP) injection of drug as indicated. All drugs were dissolved in normal saline. Each mouse underwent a 1-week washout with verification of a return to baseline SWD frequency prior to subsequent drug injection. Each mouse first received an IP injection of levetiracetam, followed by ethosuximide, phenytoin and brivaracetam. For long-term drug delivery, ethosuximide was delivered in the drinking water at a concentration of 30 mg/ml. Brivaracetam was continuously administered IP for 28 days using an osmotic minipump (ALZET Osmotic Pumps, Cupertino, CA, USA) at a rate of 8.5 mg/kg/day. Owing to the short half-life of ethosuximide in mice (1 hour) [17], periodic injections were not feasible. Minipump infusions could not be used, because the amount required exceeded the solubility of ethosuximide. Therefore, drinking water was chosen as the route of administration for ethosuximide. For brivaracetam, an osmotic minipump is the most reliable route of administration for continuous dosing.

## Results

### Transgenic Alzheimer’s disease mice have frequent epileptiform discharges

**Figure 1:**
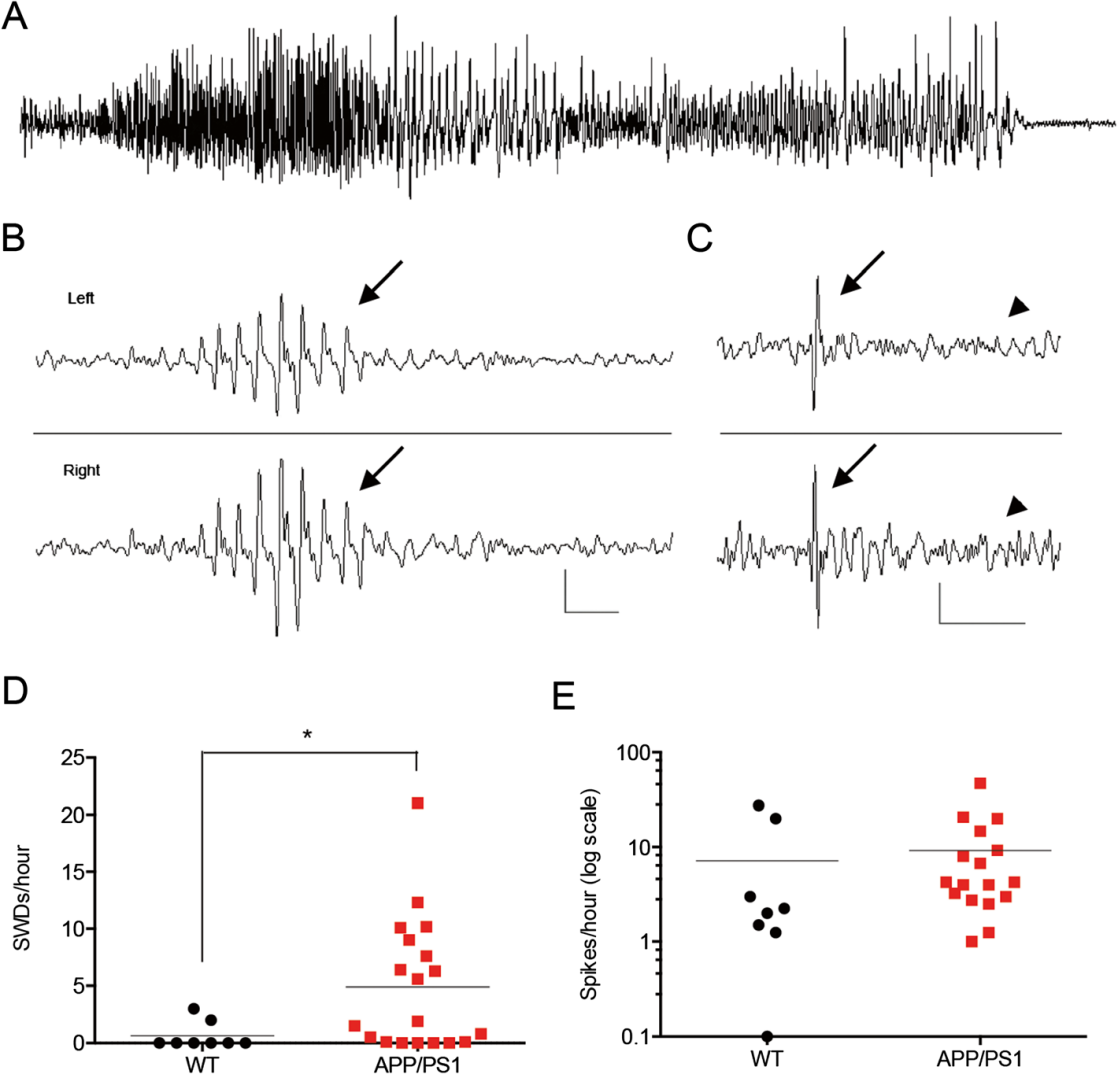
**APP/PS1 mice have frequent epileptiform discharges. (A)** Spontaneous generalized convulsive seizure, **(B)** spike-wave discharge (SWD) (arrows) and **(C)** single spike in a 10-month-old APP/PS1 mouse (arrows). The arrowheads indicate normal electroencephalogram background following single spike. **(D)** Quantification of SWDs in APP/PS1 mice compared with their wild-type (WT) littermates. **(E)** Quantification of single spikes does not show a difference between APP/PS1 mice and WT littermates (n = 8 for WT, 19 for APP/PS1). *P < 0.05 by Student’s *t*-test. WT, five females and three males; APP/PS1 mice with SWDs, four males and five females; APP/PS1 mice without SWDs, six females and four males. Calibration: SWD = 200 μV/0.5 s; spike = 200 μV/1 s.

Recently, authors have reported various types of epileptiform discharges in transgenic AD mice overexpressing APP. In the J20 model (APPswe,Ind), both generalized seizures and single spikes were reported [4], and APP/PS1 mice were found to have single spikes, clusters of SWDs and generalized seizures, when compared with nontransgenic littermate controls [5,18]. We have previously shown that 40% of aged APP/PS1 mice have convulsive seizures when recorded for 72 hours by continuous EEG video monitoring (Figure 1A) [6]. To further characterize epileptiform activity in transgenic AD mice, we assessed nineteen 8- to 10-month-old APP/PS1 mice using long-term *in vivo* EEG video monitoring. In addition to convulsive seizures, 9 (47%) of 19 APP/PS1 mice had frequent clusters of SWDs, compared with 0 of 8 of their wild-type littermates (Figure 1B,D). Using synchronized video analysis, a total of 240 hours of EEG were analyzed, and freezing behavior during the SWDs that might interfere with memory testing was quantified. Overall, only 82 SWDs were associated with brief behavioral arrest for the duration of the SWD, for a rate of less than one arrest per hour. In contrast with previous authors, we show that the frequency of single spikes is not transgene-dependent and that APP/PS1 mice do not differ from their wild-type littermates (Figure 1C,E). To assess EEG characteristics in a second AD mouse model, a limited cohort of ten 3xTg-AD mice aged 8 to 10 months underwent continuous *in vivo* EEG recording for 24 hours as described for APP/PS1 mice. None of the 3xTg-AD mice had convulsive seizures during the recording period. Four (40%) of ten of the 3xTg-AD mice had SWDs over a 12-hour period (11 ± 6 SWDs/hr). On the basis of EEG morphology, no differences in SWDs were observed between mouse strains, and the frequency of SWDs was not found to be significantly different between the two mouse models (APP/PS1 mean: 5 ± 1 SWDs/hr versus 3xTg-AD mean: 11 ± 6 SWDs/hr; *P* = 0.2 by Student’s *t*-test). Owing to the high frequency and concordance between transgenic lines, we focused on SWDs as the primary manifestation of epileptiform activity.

### Spike-wave discharges correlate with impairments in spatial memory in APP/PS1 and 3xTg-AD mice

**Figure 2:**
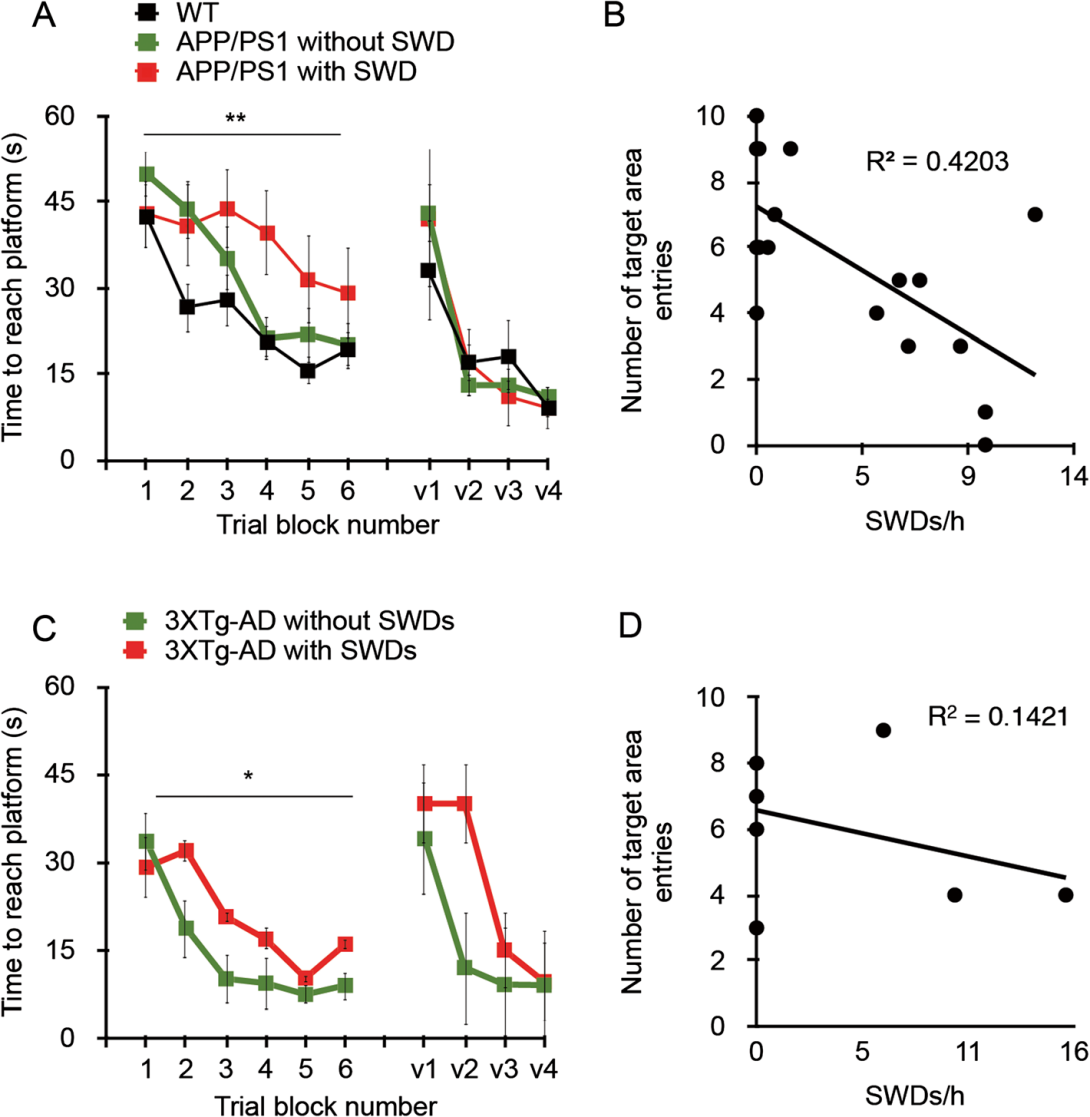
**Presence of spike-wave discharges correlates with impairments in spatial memory in APP/PS1 and 3xTg-AD mice.** Both strains of transgenic mice underwent Morris water maze testing immediately after continuous *in vivo* electroencephalography recording. **(A)** and **(C)** The presence of more than one spike-wave discharge (SWD) worsened the performance of 8- to 10-month-old APP/PS1 mice in the acquisition phase of the Morris water maze (A), and the same was true of 3xTg-AD mice (C) (*P = 0.039 and **P = 0.002 by repeated-measures analysis of variance with *post hoc* analysis). **(B)** and **(D)** A 48-hour probe trial showed an inverse relationship between frequency of SWDs and entries into the target area, defined as the platform area, during a 1-minute trial in APP/PS1 mice (B), but not in 3xTg-AD mice (D) (*P* = 0.005 and 0.39, respectively; Pearson correlation coefficient). WT: n = 8; APP/PS1 with SWDs: n = 8; APP/PS1 without SWDs: n = 9. 3xTg-AD mice included six females and four males. v1 through v4 indicate visible platform swim trials.

Although several groups have reported epileptiform discharges in AD mice, it is not known to what extent these discharges affect the phenotypic manifestations in transgenic models. We correlated the presence of SWDs in APP/PS1 and 3xTg-AD mice with spatial memory function as measured with the Morris water maze test. In APP/PS1 mice, the presence of SWDs measured prior to memory testing was associated with worsened performance in the acquisition phase of the Morris water maze (Figure 2A). Analysis of swim path length corroborated these findings (Additional file 1: Figure S1A). After 48 hours, the mice were tested for long-term memory in the probe trial. A modest inverse relationship between the number of entries in the correct target area and the number of SWDs was seen (Figure 2B). Similar findings were seen in 3xTg-AD mice, with the presence of SWDs correlating with spatial memory performance (Figure 2C). However, we did not see a correlation in the delayed probe trial between SWDs and the number of correct target entries in 3xTg-AD mice (Figure 2D), and path length analysis did not indicate a difference in memory performance of 3xTg-AD mice with SWDs compared with mice without them (Additional file 1: Figure S1D). Thus, the correlation between SWDs and spatial memory performance was less robust in 3xTg-AD transgenic mice compared with APP/PS1 mice. Although rare, SWDs can be associated with behavioral arrest that could interfere with the results of the Morris water maze test. To assess whether reduced latency to the platform observed in the transgenic AD mice with SWDs was due to excessive freezing or reduced swim speed, we measured swim speed and average resting time in addition to platform latency. The swim speed and rest times did not differ between groups (Additional file 1: Figure S1).

### Spike-wave discharges do not affect amyloid-β metabolism of plaque deposition in APP/PS1 mice

**Figure 3:**
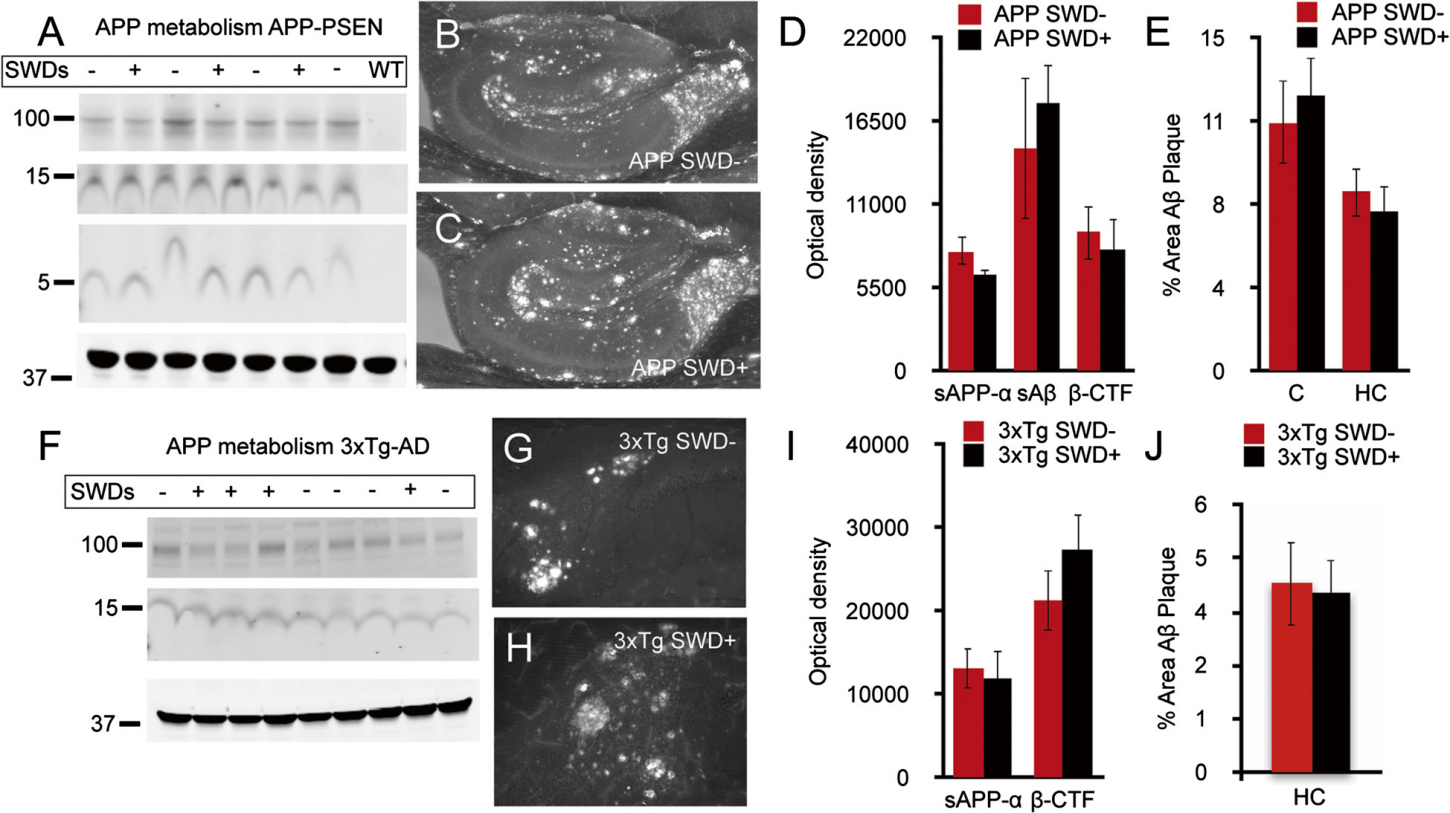
**The presence of spike wave discharges is not associated with changes in amyloid precursor protein metabolism or amyloid-β levels.** In APP/PS1 mice, the presence of spike-wave discharges (SWDs) did not alter levels of soluble amyloid precursor protein (sAPP)-α (100 kDa marker), β-C-terminal fragment (β-CTF) (15 kDa marker) or amyloid-β (Aβ) monomers (5 kDa marker) by Western blot analysis **(A, D)**. Actin loading control is indicated by the 37 kDa marker. In the same strain, Aβ plaque deposits were not altered by the presence of SWDs **(B, C, E)**. Similarly, in 3xTg-AD mice, the presence of SWDs did not alter levels of sAPP-α or β-CTF as analyzed by Western blotting **(F, I)**, nor did it alter levels of Aβ plaque **(G, H, J)**. Data were analyzed by two-tailed Student’s *t*-test. APP/PS1, n = 11; 3xTg-AD, n = 11. +Indicates the presence of SWDs, and − indicates no SWDs. HC, Hippocampus; C, Cortex; PSEN, Presenelin; WT, Wild type.

Having shown that the presence of SWDs correlates with impairments in spatial memory, we assessed whether SWDs also impact biochemical and histologic measures in transgenic AD brain, including APP metabolites and Aβ levels. Immunoblotting of soluble and detergent extracts of brain homogenates revealed no correlation between SWDs and levels of soluble APP-α, β-C-terminal fragment or Aβ in APP/PS1 or 3xTg-AD mice (Figure 3A,D,F,I). In addition, neither cortical nor hippocampal deposits of insoluble Aβ plaque differed with regard to the presence of SWDs in APP/PS1 mice (Figure 3B,C,E). Although no cortical plaques were seen in 3xTg mice at 8 to 10 months of age, the presence or absence of SWDs was not associated with hippocampal plaque density in these mice (Figure 3G,H,J). Hippocampal calbindin D-28K was first reported to be reduced in human AD several decades ago [19] and plays a role in normal hippocampal physiology as an intracellular calcium buffer [20]. Its link to epilepsy comes from the finding that patients with epilepsy have a loss of calbindin D28 in several areas of the hippocampus, and these changes have been proposed to affect the plasticity changes associated with the maintenance of the epileptic phenotype [21]. In studies in the J20 model of AD, researchers have reported a decrease in hippocampal calbindin D28 in the hippocampus, which is thought to reflect neuronal hyperexcitability [4]. In contrast to studies in J20 mice, in our present study we did not detect a reduction of calbindin D28 in APP/PS1 mice compared with WT littermates (data not shown).

### Ethosuximide and brivaracetam reduce spike-wave discharges in Alzheimer’s disease mice

**Figure 4:**
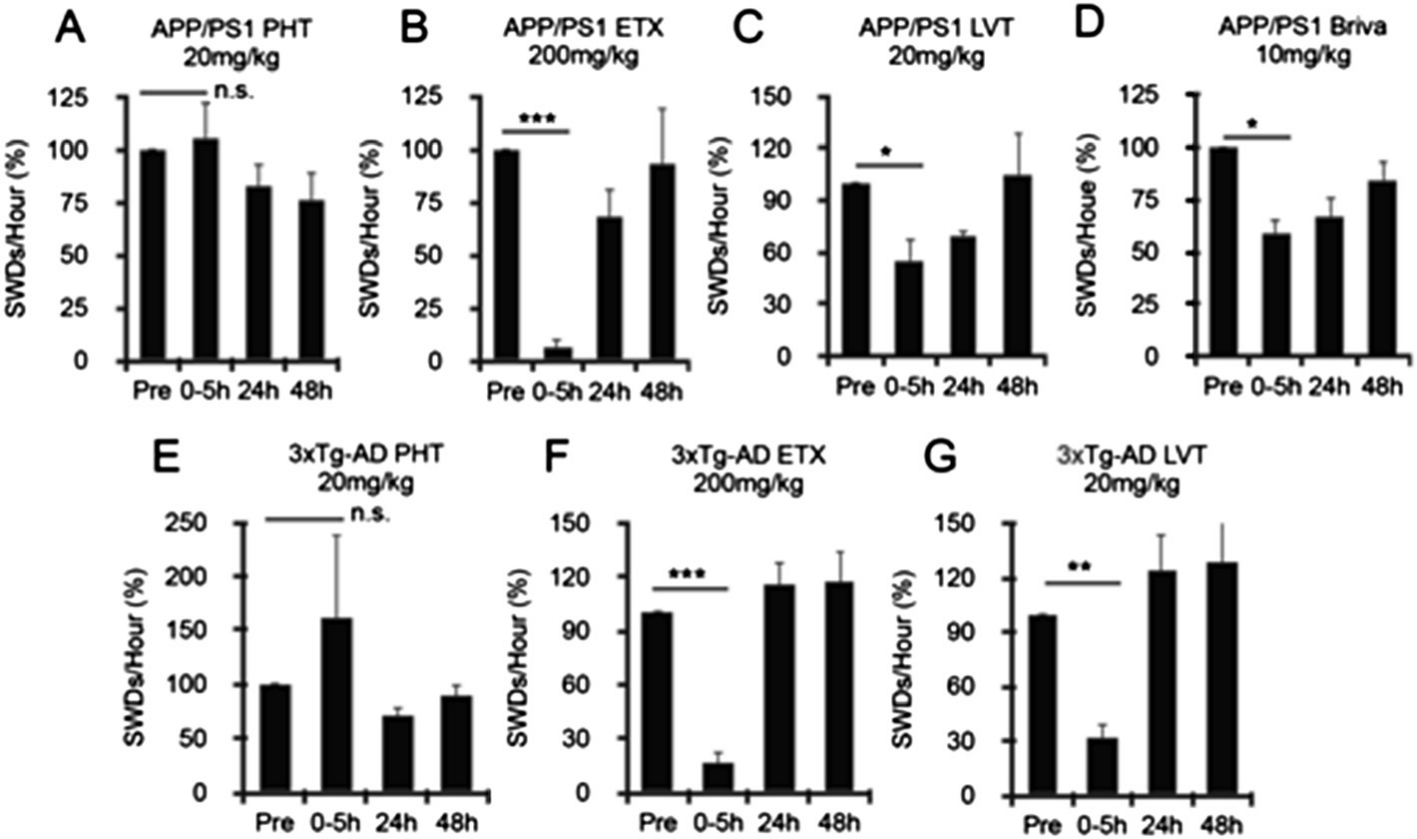
**Ethosuximide and brivaracetam reduce spike-wave discharges in transgenic Alzheimer’s disease mice.** Transgenic Alzheimer’s disease (AD) mice were given a single injection of 20 mg/kg phenytoin (PHT) **(A, E)**, 200 mg/kg ethosuximide (ETX) **(B, F)**, 20 mg/kg levetiracetam (LVT) **(C, G)** or 10 mg/kg brivaracetam (Briva) **(D)**, followed by hourly quantification of spike-wave discharges (SWDs). For phenytoin, no reduction in SWDs at 0 to 5 hours were seen in either strain (A, E) (APP/PS1: −6% ± 17 (increased SWDs), *P* = 0.75; 3xTg-AD: −61% ± 76 (increased SWDs), *P* = 0.23). Ethosuximide strongly reduced SWDs at 0 to 5 hours in both strains (B, F) (APP/PS1: 93% ± 4, ***P < 0.0001; 3xTg-AD: 83% ± 5, ***P < 0.0001). Levetiracetam reduced SWDs at 0 to 5 hours in both strains (C, G) (APP/PS1: 45% ± 12, *P < 0.01; 3xTg-AD: 61% ± 24, **P = 0.002). Brivaracetam reduced SWDs in APP/PS1 mice by 41% ± 7, * *P* < 0.01, with an *R* ^2^ of 0.95. APP/PS1, n = 4; 3xTg-AD, n = 4. *P*-values were calculated by paired two-tailed Student’s *t*-test. n.s., Not significant.

Because epileptiform discharges are relatively frequent in APP/PS1 mice and correlated with spatial memory performance, we hypothesized that the reduction of these discharges might predict the ability of anticonvulsant drugs to reverse spatial memory deficits in this mouse model. In a similar approach to that reported by Sanchez *et al*. [7], we screened several AEDs for their ability to reduce SWDs, including phenytoin, levetiracetam, brivaracetam and ethosuximide. These drugs were chosen because both phenytoin and levetiracetam have been used in AD mice previously [7,8,18] and because the SWDs reported here show similarities to ethosuximide-sensitive SWDs seen in the C3H/He mouse model of absence seizures [22]. APP/PS1 and 3xTg-AD mice at 8 to 10 months of age underwent continuous *in vivo* EEG recording for 72 hours. After a 24-hour baseline EEG, mice were given a single IP injection of drug, followed by quantification of SWDs before and after injection. In contrast to a previous report [18], phenytoin (20 mg/kg) did not acutely decrease SWDs in either APP/PS1 mice or 3xTg-AD mice (Figure 4A,E). Ethosuximide (200 mg/kg) showed the strongest reduction of SWDs in both APP/PS1 (93% ± 4) and 3xTg-AD (83% ± 5) mice, with almost complete elimination of SWDs in the first several hours after the loading dose (Figure 4B,F). Levetiracetam (20 mg/kg) reduced SWDs by 45% ± 12 in APP/PS1 mice and by 61% ±12 in 3xTg-AD mice (Figure 4C,G). Brivaracetam (10 mg/kg) reduced SWDs by 41% ± 7 in APP/PS1 mice, with a trend toward reduced frequency even at 24 hours postdose (Figure 4D).

### Brivaracetam, but not ethosuximide, reverses impairments in spatial memory in APP/PS1 mice

**Figure 5:**
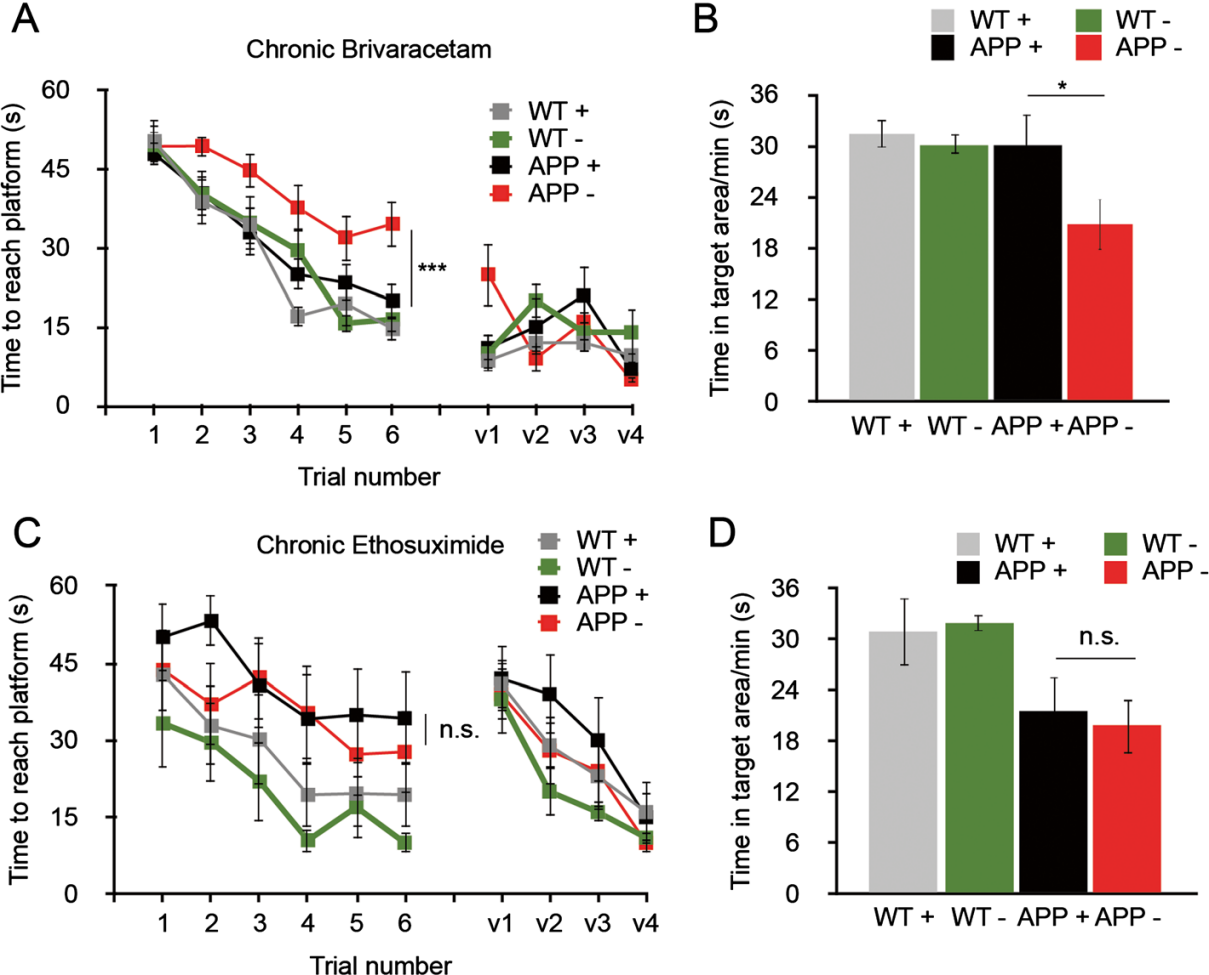
**Brivaracetam, but not ethosuximide, reverses impairments in spatial memory in APP/PS1 mice.** Aged APP/PS1 mice were administered brivaracetam by osmotic minipump or continuous delivery of ethosuximide (ETX) via drinking water. Four-week administration of brivaracetam (Briva) fully reversed memory impairments in APP/PS1 mice in the Morris water maze **(A)** and Probe Trial **(B)**. Chronic (7-week) administration of ethosuximide did not improve performance in the Morris water maze or probe trial in APP/PS1 mice **(C, D)**. For the Briva cohort, wild-type (WT) + vehicle: n = 11; WT + Briva: n = 11, APP/PS1 + vehicle: n = 15, APP/PS1 + Briva: n = 16. For chronic ETX therapy, WT + vehicle: n = 7, WT + ETX: n = 7, APP + vehicle: n = 6, APP + ETX: n = 7. *P < 0.05, ***P < 0.001, repeated-measures analysis of variance with *post hoc* comparisons. V1 through V4 indicate visible platform swim trials. + Indicates drug therapy; - indicates vehicle.

**Figure 6:**
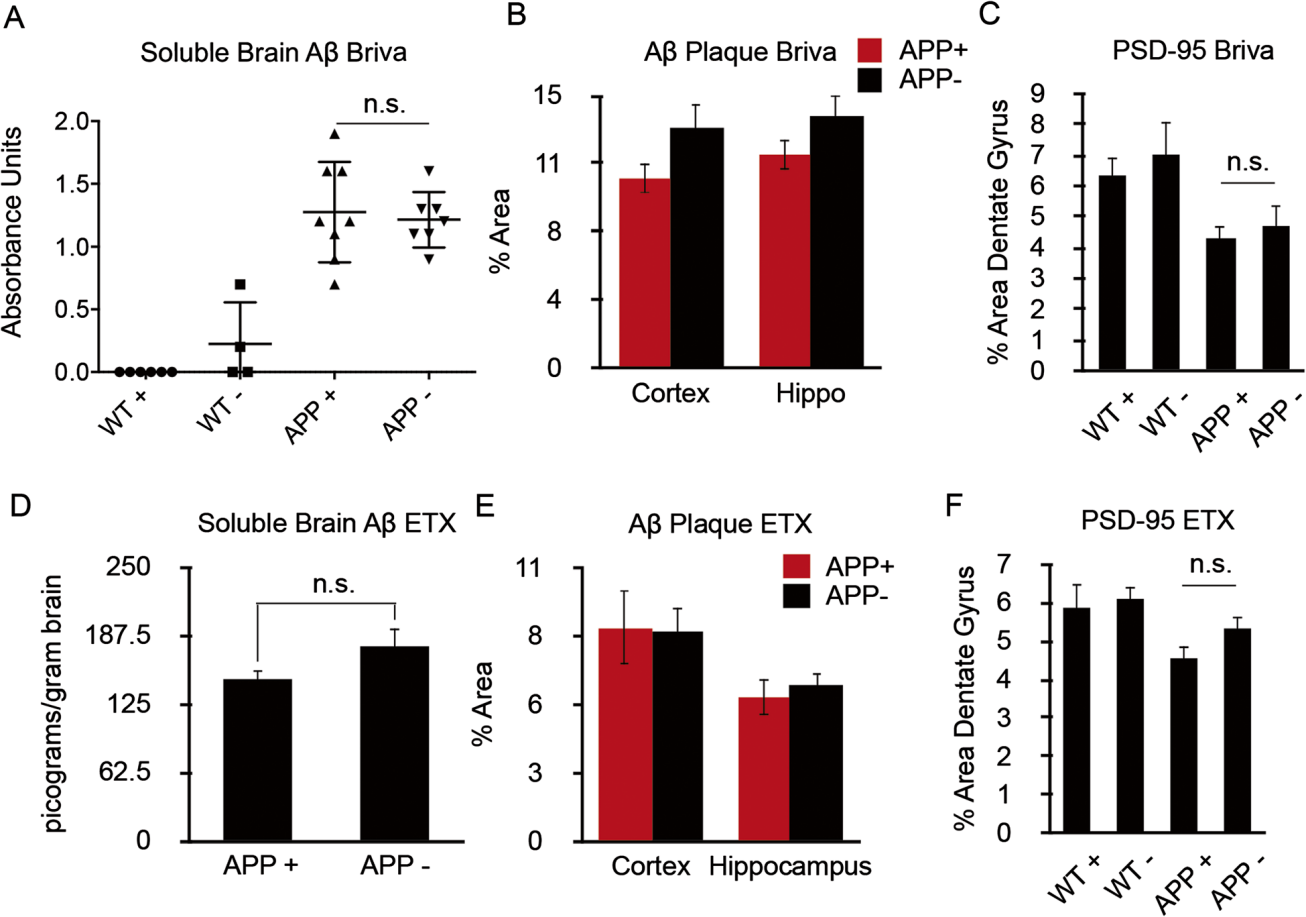
**Chronic brivaracetam or ethosuximide do not alter amyloid precursor protein metabolism or reduce synapse loss in APP/PS1 mice.** Thirteen-month old APP/PS1 mice were treated with brivaracetam (Briva) **(A–C)** or ethosuximide (ETX) **(D–F)** as in Figure 5 and then analyzed for soluble amyloid-β (Aβ) levels (A, D), Aβ plaque density (B, E) or dentate gyrus PSD-95 synaptic area (C, F). An Aβ enzyme-linked immunosorbent assay showed no effect of brivaracetam (A) or ethosuximide (D) on total Aβ monomers (*P* > 0.05 by Student’s *t*-test), and cortical and hippocampal deposits of Aβ plaque were not different between treatment groups (Student’s *t*-test) (B, E). Synaptic puncta were quantified in the molecular layer of the dentate gyrus by immunohistochemistry. Drug therapy did not reverse synapse loss seen in APP/PS1 mice (*P* > 0.05 by analysis of variance with *post hoc* comparisons). + Indicates drug therapy; - indicates vehicle. n.s., Not significant; WT, Wild type.

Having demonstrated that both brivaracetam and ethosuximide significantly reduce SWDs, we tested whether this reduction in epileptiform activity could accurately predict therapeutic reversal of impairments in spatial memory in aged APP/PS1 mice. To assess the role of brivaracetam in APP/PS1 mouse phenotypes, we treated 13-month-old mice chronically, measuring the effect of drug therapy on spatial memory, Aβ levels, synapse loss and hippocampal calbindin D28 immunoreactivity. ALZET osmotic minipumps were implanted into the APP/PS1 and WT mice, and the mice received continuous IP dosing of 8.5 mg/kg/day of brivaracetam versus saline. After 28 days, mice were tested in the Morris water maze while drug delivery was continued. Chronic brivaracetam therapy fully reversed memory impairments in APP/PS1 mice (Figure 5A,B; Additional file 2: Figure S2), but it did not change the brain concentration of soluble Aβ or insoluble plaque (Figure 6A,B). Despite the improved memory performance with brivaracetam, synapse density was not recovered (Figure 6C). Treatment with brivaracetam did not affect hippocampal calbindin D28 immunoreactivity (data not shown).

To assess the role of ethosuximide in APP/PS1 mouse phenotypes, we treated 16-month-old mice chronically (45 days), followed by measurements of spatial memory, APP metabolism and Aβ levels, synapse loss, and hippocampal calbindin D28 immunoreactivity. Drug was delivered by dissolving ethosuximide in drinking water (30 mg/ml). This dose yields a chronic plasma drug concentration of 55 μg/ml after 1 week of dosing in a separate dose range test. In the 1-week dose range test, SWDs detected by EEG were reduced from 25/hr to 6/hr, a 76% reduction. Chronic therapy with this dosage over 45 days did not reverse impairments in spatial memory in APP/PS1 mice (Figure 5C,D; Additional file 2: Figure S2), nor did this treatment affect soluble Aβ levels or Aβ plaque (Figure 6D,E). Ethosuximide did not reverse the loss of hippocampal PSD-95-positive puncta (Figure 6F) or hippocampal calbindin D28 immunoreactivity (data not shown). Thus, although both brivaracetam and ethosuximide significantly reduced SWDs in APP/PS1 mice, only brivaracetam reversed memory impairments in this model.

## Discussion

Seizures and epileptiform discharges have been observed in several strains of AD mice, including J20 and APP/PS1 transgenic models [4,6]. In the former model, seizures and single spikes have been reported, whereas the latter model also displays longer runs of epileptiform discharges resembling SWDs seen in models of absence epilepsy [18,23]. It is widely believed that seizures and epileptiform discharges play a role in the pathophysiology of AD. Chronic treatment with the anticonvulsants levetiracetam and topiramate reverses impairments in spatial memory in J20 and APP/PS1 AD mice and may affect the dynamics of both Aβ and tau protein [7,8]. Moreover, genetic knockouts found to reverse the pathologic phenotypes in AD mice also eliminate cortical hyperexcitability, including the reduction of tau protein and removal of PrP^C^ [6,11]. A low dose of levetiracetam was recently shown to reduce hippocampal hyperactivity during encoding processes in patients with aMCI [9]. This reduction showed slight improvements in select hippocampal function, suggesting that neuronal hyperactivity in aMCI may be a pathologic rather than compensatory response to neurodegeneration and reduced connectivity. Thus, accumulating indirect evidence suggests that cortical hyperactivity may play an important role in the pathophysiology of AD, making chronic EEG recordings a promising marker of target engagement and efficacy for new drugs for AD.

We found that SWDs constitute the most frequent epileptiform discharges in APP/PS1 mice, in contrast to the J20 mice, in which single spikes seem to predominate [4]. SWDs correlate with worsened memory performance in APP/PS1 mice, which we considered as a promising feature for a possible surrogate marker of both disease and drug efficacy. However, the pharmacologic elimination of SWDs does not consistently predict improvements in spatial memory. Indeed, although both ethosuximide and brivaracetam significantly reduced SWDs in APP/PS1 mice, only the latter reversed impairments in spatial memory performance in these mice. These findings suggest that a reduction in SWDs does not represent a robust surrogate marker of drug efficacy in APP/PS1 mice. Our data further emphasize the role of drugs targeting SV2A, such as levetiracetam and brivaracetam, in reversing spatial memory impairments across several AD mouse strains [7]. Further, our conclusion that chronic ethosuximide administration does not reverse memory impairments in APP/PS1 mice is important as, apart from its antiepileptic effects, ethosuximide has previously been shown to have neuroprotective properties and thus is seen as a candidate to alleviate aging and age-related disease. In a screen of compounds that affect longevity in the *Caenorhabditis elegans* model, ethosuximide was found to extend lifespan by an average of 17% [24]. The underlying mechanism was later found to be modulation of sensory perception by ethosuximide with reduced sensorineural activity [25]. Ethosuximide has also been shown to prevent cochlear injury in a mouse model of sensorineural hearing loss, again linked to reducing neuronal activity [26]. Despite these interesting findings, ethosuximide does not appear to have a therapeutic effect in the APP/PS1 model of AD.

Several limitations of our data must be considered. We focused on SWDs as these are the most frequent epileptiform discharges unique to APP/PS1 mice compared with their nontransgenic littermates. However, it is not yet known which, if any, epileptiform discharges predominate in patients with AD. We note that Sanchez *et al*. [7] reported single spikes as the predominant epileptiform activity in the J20 mouse model of AD. Although it is likely these findings represent strain differences, their importance in AD pathophysiology is unclear. Seizures have been studied for decades in humans with AD, but the presence of epileptiform discharges, which requires EEG recordings, are not well characterized. In the largest study to date, researchers examined routine EEG recordings from 1,674 patients with various cognitive disorders, including 510 with AD and 225 with MCI [27]. Of the former, 2% had epileptiform discharges on routine EEG, the same as the percentage seen among patients with “subjective complaints.” There was no correlation between the presence of epileptiform discharges and performance on bedside neuropsychological testing, and it was concluded that routine EEG could not be recommended as part of routine clinical workup in AD. In another study, the investigators reported a frequency of epileptiform discharges of 16% in patients with AD [2]. In a more recent report, authors showed a frequency of 62% in aMCI and AD patients known to have epilepsy and 6% in patients without seizures [28]. The presence of epileptiform discharges also predicted earlier cognitive decline [28]. None of the studies published to date have differentiated various types of epileptiform discharges, thus limiting the correlation to SWDs reported here. The current evidence would suggest that epileptiform activity is less prominent in sporadic AD than in mouse models of autosomal dominant disease. However, prospective studies with long-term EEG monitoring are needed to further characterize cortical hyperexcitability in AD, the relationship of EEG profiles to AD pathophysiology, and whether the presence and reduction of epileptiform discharges may represent a marker of drug efficacy.

Our primary objective was to establish whether epileptiform discharges could be used as a marker for overall drug efficacy in improving memory function in an AD mouse model. Thus, we did not test whether a subgroup of mice, all displaying SWDs, would respond better to ethosuximide therapy compared with a mixed group with varying SWD frequencies. Our findings do suggest that a reduction in SWDs is not sufficient to reverse memory impairments in APP/PS1 mice, but future studies using a different experimental design are required to extend the generalizability of this finding. We also note that both J20 and APP/PS1 mice have prominent epilepsy, and our divergent findings with ethosuximide and brivaracetam with respect to reversal of impairments in spatial memory may be explained by the efficacy of treating partial versus generalized seizures. Ethosuximide is exclusively used for absence seizures in humans, with a narrow antiepileptic spectrum, whereas brivaracetam has broad antiepileptic action.

## Conclusions

Our study is the first to demonstrate efficacy of brivaracetam in treating impairments in spatial memory in AD mice. Brivaracetam interacts with SV2A, and is closely related to the widely used anticonvulsant levetiracetam. As noted, two previous studies in J20 and APP/PS1 mice have shown clear benefits of levetiracetam in reversing memory impairments in this model, suggesting that targeting SV2A alleviates AD symptoms across AD models. We also show that, despite some promise as a neuroprotective agent in other model systems, chronic ethosuximide treatment does not reverse impairments in spatial memory in APP/PS1 mice. Moreover, whereas SWDs in APP/PS1 mice correlate with impairments in spatial memory, the reduction of these discharges is not a reliable surrogate marker of preclinical drug efficacy in the APP/PS1 AD mouse model.

## Additional files

Additional file 1: Figure S1. Extended Morris water maze analysis of APP/PS1 and 3xTg-AD mice. Path length, average swim speed and time spent resting were analyzed as described for platform latency in Figure 2. The presence of >1 SWDs worsened performance of 8- to 10-month-old APP/PS1 mice in the acquisition phase of the Morris water maze using path length analysis **(A)** (**P* < 0.05 by repeated-measures ANOVA with least significant difference *post hoc* analysis). Path lengths were not different in 3xTg-AD mice with or without SWDs **(D)**. Swim speed and time spent resting were even across mouse cohorts **(B**, **C**, **E**, **F)**. Additional file 2: Figure S2. Extended Morris water maze analysis of APP/PS1-treated mice with chronic brivaracetam or ethosuximide. Path length, average swim speed and time spent resting were analyzed as described for platform latency in Figure 5. Path length was significantly shorter in APP/PS1 mice treated with brivaracetam compared with APP/PS1 mice that were on vehicle therapy **(A)** (*P* < 0.001 by repeated-measures ANOVA with *post hoc* comparisons). Chronic ethosuximide treatment did not alter path length in the Morris water maze **(D)**. Neither brivaracetam nor ethosuximide treatment affected average swim speed or average resting time across mouse cohorts **(B**, **C**, **E**, **F)**. + Indicates drug therapy; - indicates vehicle.

## Abbreviations

Aβ: Amyloid-β
AD: Alzheimer’s disease
AED: Antiepileptic drug
aMCI: Amnestic mild cognitive impairment
ANOVA: Analysis of variance
APP: Amyloid precursor protein
β-CTF: β-C-terminal fragment
EEG: Electroencephalography
ELISA: Enzyme-linked immunosorbent assay
ETX: Ethosuximide
IP: Intraperitoneal
PBS: Phosphate-buffered saline
PFA: Paraformaldehyde
PHT: Phenytoin
PrP^C^: Cellular prion protein
SWD: Spike-wave discharge
SV2A: Synaptic vesicle protein 2A
TBS: 50 mM Tris-HCl, 150 mM NaCl
TBST: Tris-buffered saline and Tween 20 mixture
