# Supplementary figures and images for: Brivaracetam, but not ethosuximide, reverses memory impairments in an Alzheimer’s disease mouse model
Source: Alzheimers Res Ther. 2015 May 5;7(1):25. doi: 10.1186/s13195-015-0110-9 (PMC4419386; doi:10.1186/s13195-015-0110-9)

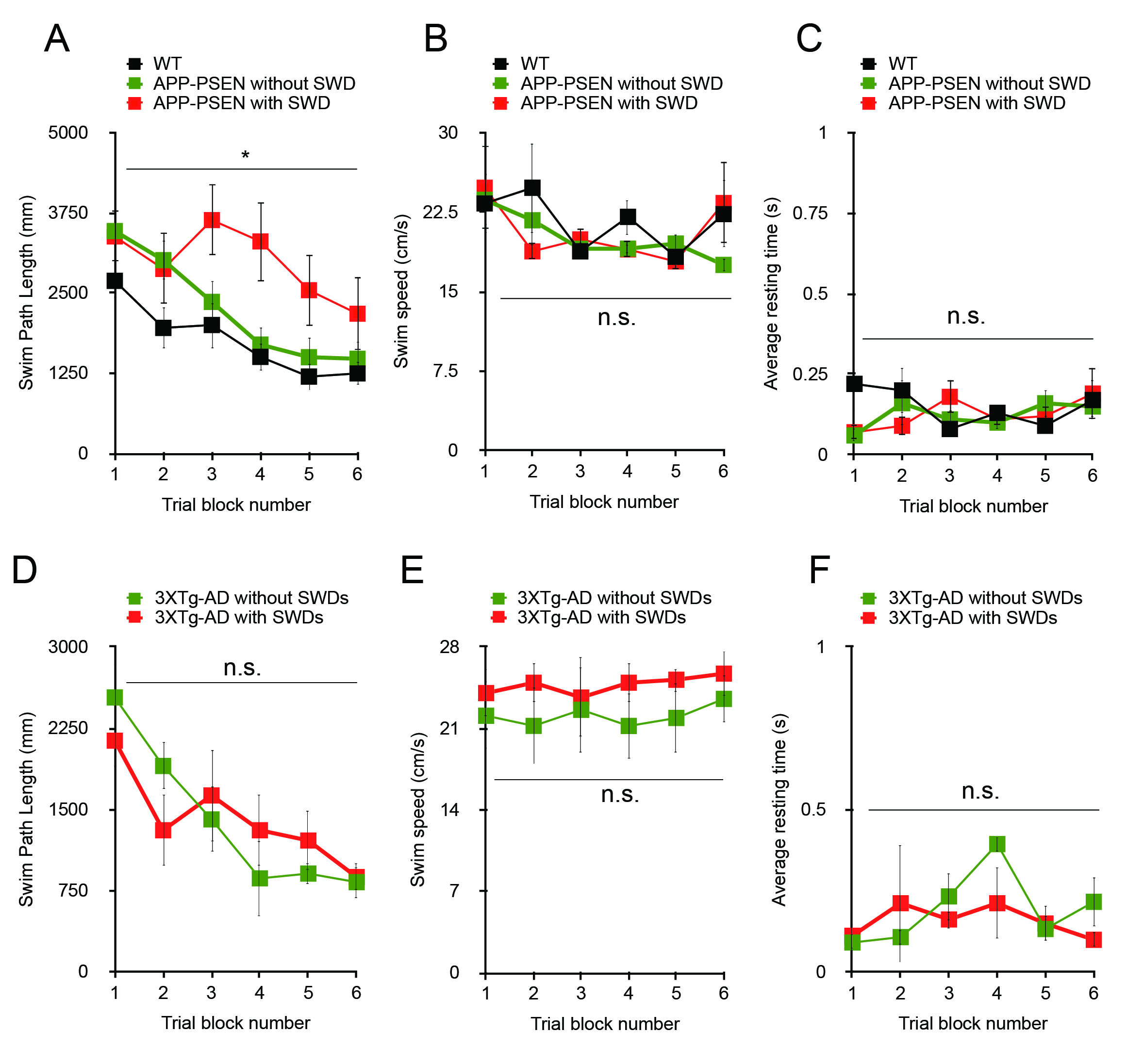

Supplement: Additional file 1: Figure S1. — Extended Morris water maze analysis of APP/PS1 and 3xTg-AD mice. Path length, average swim speed and time spent resting were analyzed as described for platform latency in Figure 2. The presence of >1 SWDs worsened performance of 8- to 10-month-old APP/PS1 mice in the acquisition phase of the Morris water maze using path length analysis (A) (*P < 0.05 by repeated-measures ANOVA with least significant difference post hoc analysis). Path lengths were not different in 3xTg-AD mice with or without SWDs (D). Swim speed and time spent resting were even across mouse cohorts (B, C, E, F). [file 13195_2015_110_MOESM1_ESM.tiff]

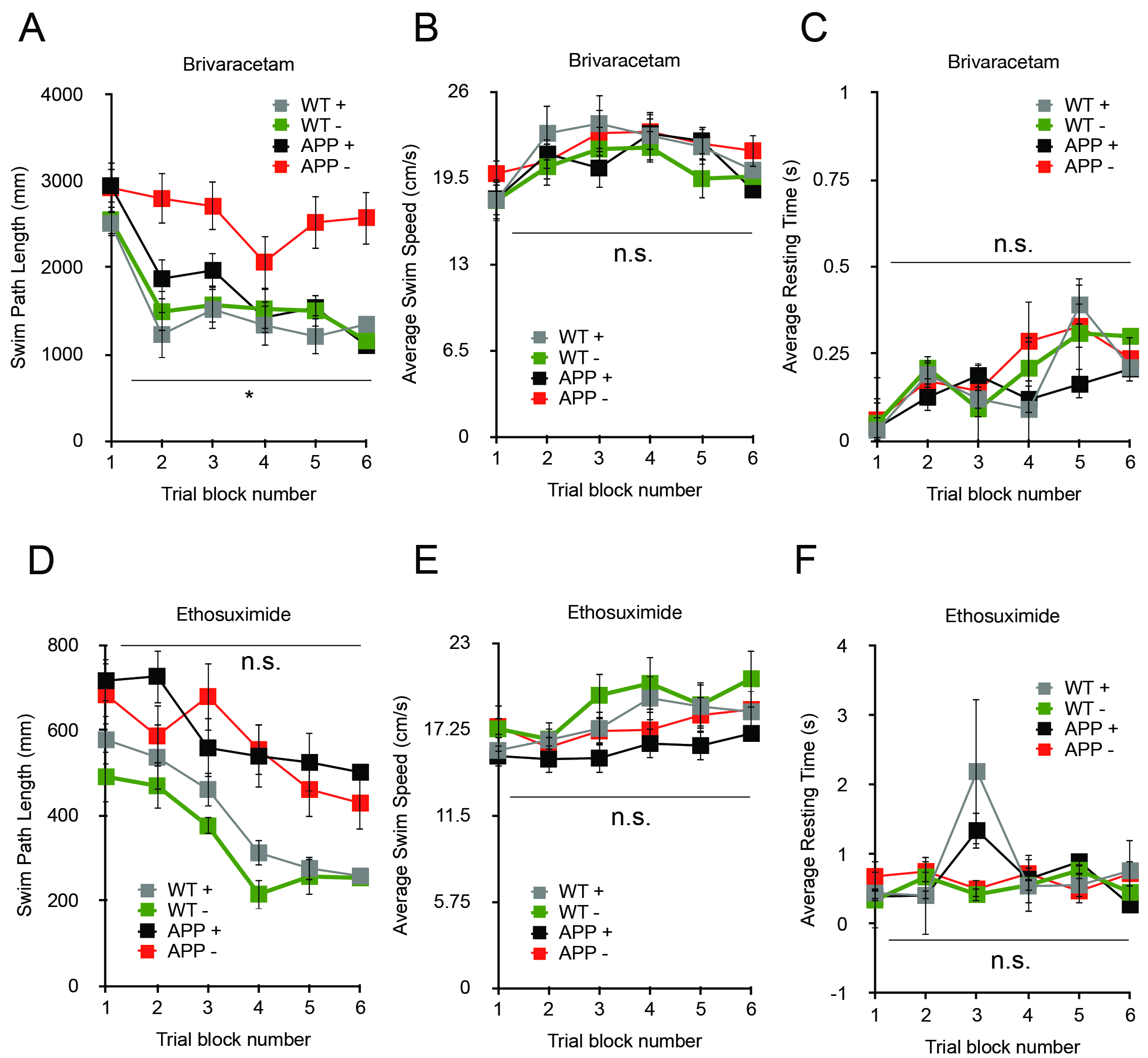

Supplement: Additional file 2: Figure S2. — Extended Morris water maze analysis of APP/PS1-treated mice with chronic brivaracetam or ethosuximide. Path length, average swim speed and time spent resting were analyzed as described for platform latency in Figure 5. Path length was significantly shorter in APP/PS1 mice treated with brivaracetam compared with APP/PS1 mice that were on vehicle therapy (A) (P < 0.001 by repeated-measures ANOVA with post hoc comparisons). Chronic ethosuximide treatment did not alter path length in the Morris water maze (D). Neither brivaracetam nor ethosuximide treatment affected average swim speed or average resting time across mouse cohorts (B, C, E, F). + Indicates drug therapy; - indicates vehicle. [file 13195_2015_110_MOESM2_ESM.tiff]
